# Capture threshold of bipolar and unipolar pacing of left ventricle via coronary sinus branch: longitudinal study

**DOI:** 10.3389/fcvm.2023.1096538

**Published:** 2023-05-23

**Authors:** Jakub Stritecky, Jan Kremlacek, Josef Hanus, Ludek Haman, Hana Stritecka, Jakub Simka, Petr Parizek

**Affiliations:** ^1^ Department of Medical Biophysics, Faculty of Medicine in Hradec Kralove, Charles University, Hradec Králové, Czech Republic; ^2^ 1st Department of Internal Medicine – Cardioangiology, University Hospital Hradec Kralove, Hradec Kralove, Czech Republic; ^3^ Department of Military Internal Medicine and Military Hygiene, Faculty of Health Sciences, University of Defence, Hradec Králové, Czech Republic

**Keywords:** quadripolar lead, pacing capture threshold energy, pacing polarity, steroid eluting lead, resynchronisation therapy, battery longevity

## Abstract

**Introduction:**

The aim of this paper is to first monitor the changes in the capture threshold of endovascularly placed leads for left ventricle pacing, second to compare the pacing configurations, and third to verify the effect of Steroid elution for endovascular leads.

**Sample and Method:**

The study included 202 consecutive single centre patients implanted with the Quartet™ lead (St. Jude Medical). The capture threshold and related lead parameters were tested during implantation, on the day of the patient’s discharge, and 3, 9, and 15 months after implantation. The electrical energy corresponding to the threshold values for inducing ventricular contraction was recorded for subgroups of patients with bipolar and pseudo-unipolar pacing vectors and electrodes equipped with and without a slow-eluting steroids. The best setting for the resynchronization effect was generally chosen. Capture threshold was taken as a selection criterion only if there were multiple options with (expected) similar resynchronization effect.

**Results and Discussion:**

The measurements showed that the ratio of threshold energies of UNI vs. BI was 5× higher (*p* < 0.001) at implantation. At the end of the follow-up, it dropped to 2.6 (*p* = 0.012). The steroid effect in BI vectors was caused by a double capture threshold in the NSE group compared to the SE group (*p* < 0.001), increased by approximately 2.5 times (*p* < 0.001). The study concludes that after a larger initial increase in the capture threshold, the leads showed a gradual increase in the entire set. As a result, the bipolar threshold energies increase, and the pseudo-unipolar energies decrease. Since bipolar vectors require a significantly lower pacing energy, battery life of the implanted device would improve. When evaluating the steroid elution of bipolar vectors, we observe a significant positive effect of a gradual increase of the threshold energy.

## Introduction

Cardiac pacing is a common method of treating bradyarrhythmias, but also proven in the treatment of heart failure (1). Non-pharmacological treatment of heart failure is implemented by resynchronization therapy, which uses, in addition to a lead located in the right ventricle, a lead located on the left ventricle lateral wall.

For the left ventricular pacing, either bipolar or pseudo-unipolar vectors can be used. Bipolar vectors are defined by pacing both electrodes on the same lead. The Pseudo-unipolar vectors’ cathode is the electrode on the left ventricular lead and the anode is either the defibrillation coil of the lead located in the right heart chamber, the ring of the right ventricular pacing lead or the can of the implanted device.

Some pacing electrodes may be equipped with a slow-release, locally acting steroid to minimize tissue damage at the lead placement site. The Quartet™ lead is equipped with a steroid only on the distal electrode.

During the implant procedure and outpatient follow-ups, the pacing’s parameters are routinely monitored and the capture threshold is tested. The capture threshold changes as a result of healing processes, mineral imbalances or applications of certain drugs. Therefore, to ensure effective pacing, it is necessary to set a sufficient reserve in the pacing output.

Unlike the development of the capture threshold of an endocardial lead with active or passive fixation (2, 3), which is well documented, the progression and stability of the capture threshold of endovascularly placed leads (4) is almost unreported in the literature especially with impact on comparing bipolar x unipolar and steroid x steroid-free vectors. This motivated the monitoring and course of the capture threshold of an endovascular electrode, specifically Quartet™ (St. Jude Medical), implanted in the venous system of the left heart ventricle.

### Sample

The study included 202 consecutive single centre patients: 160 men and 42 women diagnosed with an ischemic cardiomyopathy (iCM), non-ischemic cardiomyopathy (CM) or a combination of both (Table 1).

**Table 1:** Characteristics of the group, distribution by gender, cardiomyopathy (CM), and ischemic cardiomyopathy (iCM).

	*n*	Age	iCM	CM	iCM + CM
Male	160 79.2%	69.6 ± 7.5 years	99 61.9%	47 29.4%	14 8.8%
Female	42 20.8%	69.1 ± 8.6 years	14 33.3%	27 64.3%	1 2.4%
Total	202 100%	69.5 ± 7.7 years	113 55.9%	74 36.6%	15 7.4%

Based on the current practice, patients were indicated for resynchronization therapy (5). Most of the patients suffered from a complete Left bundle branch block—“true LBBB” (6)—86 (43.4%). The second largest group comprised of patients with LBBB-type activation disorder—62 (31.3%). Other patients were indicated for elective catheter ablation of the AV junction or suffered from symptomatic bradycardia, qualifying them for ventricular pacing.

Patients with anatomical or technical reasons that ruled out the use of a quadripolar left ventricular electrode were excluded. Furthermore, all patients undergoing lead repositioning or pacing vector change during the follow-up were also excluded.

Based on current practice, all patients were discharged with a high pacing percentage due to effective resynchronization therapy.

The sample of 202 (100%) patients was divided according to the pacing vector into bipolar (BI) and pseudo-unipolar (UNI), where the bipolar vector was used in 171 (84.7%) and pseudo-unipolar vector in 31 (15.3%) patients.

The group of patients with bipolar vectors 171 (100%) was further divided into subgroups with the support of a locally released steroid (SE) 49 (28.7%) and without it (NSE) 122 (71.3%) (Table 2).

**Table 2:** Individual controls and energies of threshold pacing pulses first for the whole set then with a division into pseudo-unipolar and bipolar vectors (BI x UNI) and finally according to whether they are enhanced on their cathode with a slow-release Steroid (SE x NSE).

	Days from implant	*n*	Energy (µJ) (95% CI)		*n*	Energy (µJ) (95% CI)		*n*	Energy (µJ)
	(min;max)								(95% CI)
IMPLANT	0	202	0.73 [0.374; 2.045]	BI	171	0.658 [0.356; 1.467]	NSE	122	0.690 [0.412; 1.753]
							SE	49	0.377 [0.164; 0.900]
				UNI	31	Mar-19	NSE	24	3.319 [0.865; 5.652]
						[0.961; 7.743]	SE	7	2.500 [1.495; 19.069]
Follow-up1	1.2 ± 0.7 (1;5)	200	1.046 [0.454; 3.014]	BI	169	0.859	NSE	120	1.059 [0.601; 3.072]
						[0.403; 2.000]	SE	49	0.365 [0.186; 1.050]
				UNI	31	Mar-48	NSE	24	3.441 [1.939; 8.166]
						[1.708; 11.249]	SE	7	11.484 [1.680; 15.633]
Follow-up2	92.1 ± 23.5 (2;192)	195	1.098 [0.524; 2.693]	BI	165	0.974	NSE	118	1.363 [0.771; 2.500]
						[0.476; 2.125]	SE	47	0.465 [0.304; 0.841]
				UNI	30	4.288	NSE	23	4.201 [1.511; 8.508]
						[1.169; 11.139]	SE	7	13.636 [0.918; 18.616]
Follow-up3	278.6 ± 51.4 (66;458)	189	1.361 [0.646; 2.857]	BI	160	1.231	NSE	115	1.494 [0.900; 2.860]
						[0.614; 2.248]	SE	45	0.600 [0.415; 0.878]
				UNI	29	4.287	NSE	22	4.153 [1.396; 7.637]
						[1.063; 8.813]	SE	7	10.246 [0.703; 17.365]
Follow-up4	475.9 ± 58.4 (248;679)	182	1.307 [0.630; 2.947]	BI	154	1.238	NSE	112	1.593 [0.898; 2.805]
						[0.619; 2.365]	SE	42	0.632 [0.426; 1.231]
				UNI	28	3.297	NSE	22	3.297 [0.855; 8.732]
						[0.789; 9.568]	SE	6	6.418 [0.413; 18.238]

## Method

The study was observational. No special rules were applied for setting up the implanted devices. For all indicated patients, three shapes of the Quartet™ lead (St. Jude Medical; 1456Q, 1458Q, 1458QL) were used. Using the LVCap™ Confirm function, measurements were performed automatically during all five scheduled follow-ups:

1. during implantation (IMPL)
2. first follow-up at discharge (usually the second day after implantation) (FU1)
3. second follow-up after 3 ± 1 months from implantation (FU2)
4. third follow-up after 9 ± 2 months from implantation (FU3)
5. fourth follow-up after 15 ± 2 months from implantation (FU4)

The total follow-up period reached approximately 15 ± 2 months. Five values of the were taken. The threshold is defined as the minimum amount of energy needed to capture the myocardial tissue electrically.

The LVCap™ Confirm function (St. Jude Medical) determines the measurement accuracy, which is 1/8 V with a given pulse width (0.1 ms resolution). For comparability of the capture thresholds at different pulse widths, the pulse energy was calculated using the formula E = (U^2^ * t)/R (7). The pacing impedance was measured by the device with an accuracy of 1Ω. All energies are given in µJ.

**Figure 1:**
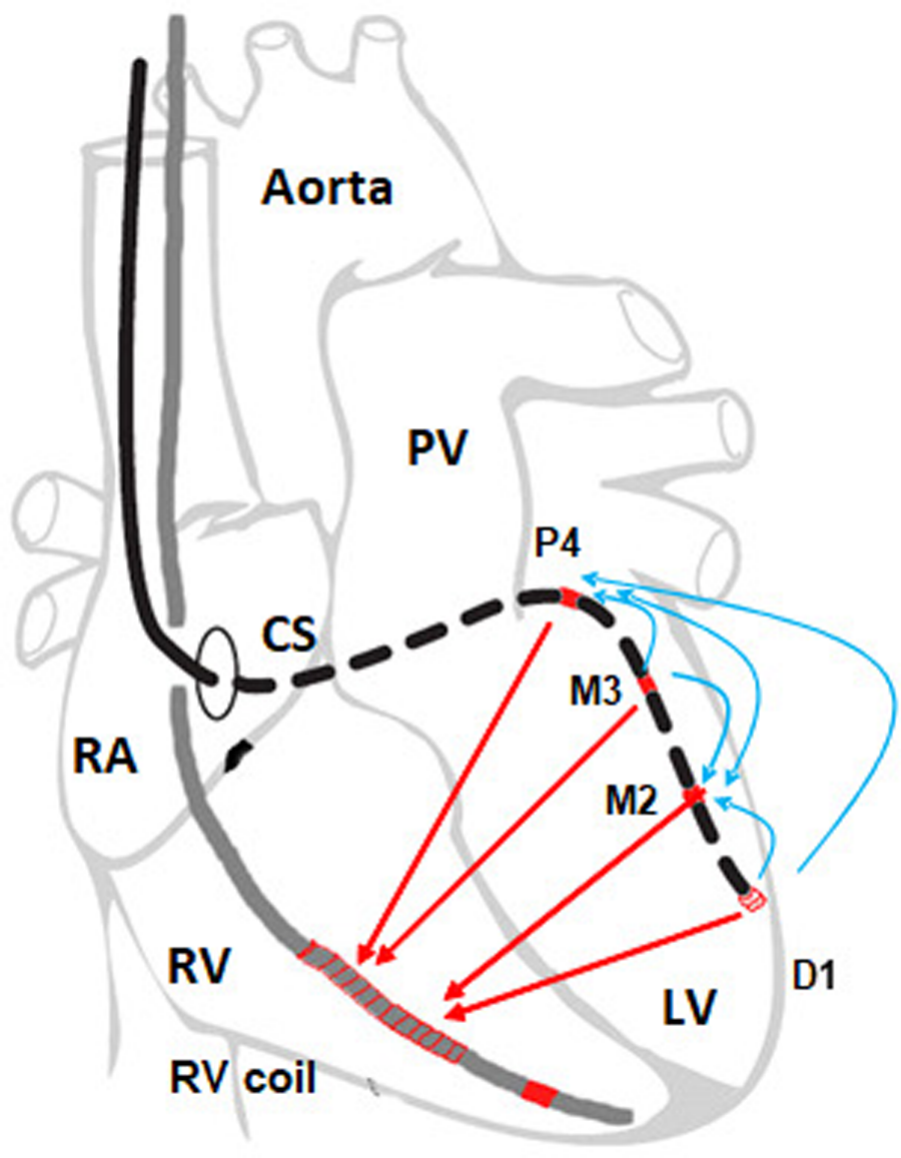
Pacing vectors from the left ventricular lead in the resynchronization system. The vectors between electrodes D1, M2, M3 and P4 (shown in blue) are marked as bipolar, and those that have an RV coil as an anode (shown in red) are called pseudo-unipolar. Steroid coverage is only on D1, therefore D1-X vectors are marked as *steroid*.

The four LV lead electrodes (Distal to Proximal: D1, M2, M3, P4—based on company terminology) utilized as potential LV pacing cathodes, with M2, P4, and the coil of the RV electrode as potential anodes. Six bipolar LV pulse vectors (D1-M2, D1-P4, M2-P4, M3-M2, M3-P4 and P4-M2) and four pseudo-unipolar (D1-RVcoil, M2-RVcoil, M3-RVcoil, P4-RVcoil) vectors were defined as a cathode-anode and presented on Figure 1.

All measurements were performed on implantable defibrillators that allow pseudo-unipolar pacing only against the coil of the defibrillation lead.

Steroid-eluting vectors have as their cathode the distal electrode pole of the lead marked D1.

In the statistical evaluation of the set, tests of normality and homogeneity of variances rule out the subsequent use of parametric tests, even after logarithmic transformation. Therefore, non-parametric tests were used, and descriptive statistics are reported as median and lower and upper quartile. During the evaluation, the hypotheses tested whether the factor of connection or steroid-eluting of the electrode has an effect on the total electrical energy of the effective pacemaker pulse. Furthermore, the stability of these parameters in the post-implantation phase (3–15 months) was evaluated.

To evaluate the course of the threshold energy over the time measured, the Friedman test, a non-parametric variant of the analysis of variance, was used for repeated measurements. In the case of a statistically significant result, Dubin-Conover pairwise comparisons were subsequently used. The non-parametric Mann–Whitney *U* test was used to evaluate the difference between patients (BI-UNI, SE-NSE comparison).

## Results

Patients' capture thresholds were scheduled to be taken first during implantation, second at discharge, and then after 3, 9, and 15 months (see Table 2). The patient visit schedule was designed according to the standard procedures of the department.

Mean values of threshold energy and quartiles at individual controls and their distribution according to modalities are shown in Table 2. The threshold energy was calculated based on the formula E = (U^2^ * t)/R.

Decreasing follow-up totals account for patient termination of the follow-ups prior to the study termination. The reason for the termination of the follow-ups was patient death or heart transplantation. If there was repositioning or a change of the lead or a change of the pacing vector, the patient was excluded from the study.

### Sample statistics

For the entire group of patients, the statistical significance of changes in pacing energies between individual visits was tested using the Friedman test (*p* < 0.001). Pairwise comparisons were performed for individual visits and calculated to be statistically significantly different. Values after implantation (IMPL) increased by approximately 43% (*p* < 0.001) to discharge (FU1). By the first visit after stabilization of the parameters (FU2), there was an insignificant increase of 5% (*p* = 0.321). The increase in threshold energy from implantation values (IMPL) to steady-state values (FU2) was a total of 50% (*p* < 0.001). In the next follow-up, there was a gradual statistically significant increase in threshold energies by 19% from FU2 (*p* = 0.045). The overall increase in capture threshold energy from implantation to the fifth final control (FU4) was 80% with strong statistical significance (*p* < 0.001) (Tables 2, 3).

**Table 3:** Changes in pacing thresholds between measurements.

Pairwise Comparisons (Durbin-Conover)										
			Part A		Part B		Part C		Part D	
			Whole sample		Bipolar vectors (BI)		Bipolar Steroid-eluted vectors (UNI)		Bipolární vektory se steroidem (BI, Ster)	
			Difference Mean [95% CI]	*p*	Difference Mean [95% CI]	*p*	Difference Mean [95% CI]	*p*	Difference Mean [95% CI]	*p*
IMPL	–	FU1	0.173 [0.495; 1.263]	**<.001**	0.153 [0.377; 0.887]	**<.001**	0.369 [0.140; 4.311]	**0**.**011**	0.000 [−0.162; 0.118]	0.748
IMPL	–	FU2	0.237 [0.324; 1.449]	**<.001**	0.237 [0.231; 0.875]	**<.001**	0.211 [−0.563; 6.003]	**0**.**024**	0.018 [−0.243; 0.331]	0.372
IMPL	–	FU3	0.413 [0.306; 1.165]	**<.001**	0.450 [0.440; 1.106]	**<.001**	0.176 [−1.695; 2.751]	0.196	0.186 [−0.136; 0.392]	**0**.**018**
IMPL	–	FU4	0.426 [0.325; 1.358]	**<.001**	0.408 [0.427; 1.162]	**<.001**	0.596 [−1.728; 3.936]	0.463	0.226 [−0.098; 0.562]	**0**.**007**
FU1	–	FU2	0.048 [−0.594; 0.559]	0.321	0.072 [−0.464; 0.279]	0.219	−0.176 [−2.913; 3.694]	0.762	0.073 [−0.219; 0.343]	0.226
FU1	–	FU3	0.155 [−0.737; 0.416]	**0.001**	0.219 [−0.211; 0.466]	**<.001**	−0.900 [−5.124; 1.624]	0.196	0.179 [−0.085; 0.372]	**0**.**007**
FU1	–	FU4	0.199 [−0.719; 0.592]	**0.003**	0.241 [−0.194; 0.510]	**<.001**	−0.305 [−5.257; 2.692]	0.065	0.228 [−0.030; 0.536]	**0**.**002**
FU2	–	FU3	0.120 [−0.864; 0.636]	**0.027**	0.144 [0.021; 0.549]	**0**.**004**	−0.041 [−7.145; 2.514]	0.321	0.120 [−0.064; 0.286]	0.135
FU2	–	FU4	0.131 [−0.892; 0.774]	**0.045**	1.190 [−0.057; 0.606]	**0**.**004**	−0.175 [−7.217; 3.434]	0.122	0.154 [−0.086; 0.532]	0.065
FU3	–	FU4	−0.015 [−0.201; 0.339]	0.834	0.000 [−0.214; 0.193]	0.985	−0.130 [−0.911; 1.925]	0.574	0.031 [−0.085; 0.316]	0.721
			Part E		Part F		Part G			
			Bipolar steroid-free vectors (BI, NoSter)		Pseudo-unipolar steroid-eluted vectors (UNI, Ster)		Pseudo-unipolar Steroid-free vektors (UNI, NoSter)			
			Difference Mean [95% CI]	*p*	Difference Mean [95% CI]	*p*	Difference Mean [95% CI]	*p*		
IMPL	–	FU1	0.272 [0.555; 1.124]	**<.001**	−0.043 [−3.446; 6.032]	0.736	0.710 [0.000; 4.995]	**0**.**007**		
IMPL	–	FU2	0.472 [0.323; 1.188]	**<.001**	−1.141 [−4.120; 6.048]	0.736	0.700 [−0.902; 7.410]	**0**.**018**		
IMPL	–	FU3	0.706 [0.580; 1.471]	**<.001**	−0.149 [−4.601; 7.512]	0.866	0.212 [−2.310; 2.777]	0.119		
IMPL	–	FU4	0.683 [0.518; 1.492]	**<.001**	−0.191 [−7.975; 15.974]	0.614	0.734 [−2.292; 2.927]	0.26		
FU1	–	FU2	0.071 [−0.663; 0.356]	0.492	−0.157 [−4.428; 3.772]	1.000	−0.195 [−3.662; 4.882]	0.731		
FU1	–	FU3	0.286 [−0.344; 0.587]	**0.002**	−0.649 [−8.434; 8.759]	0.614	−0.935 [−6.290; 1.572]	0.24		
FU1	–	FU4	0.245 [−0.352; 0.597]	**0.003**	−0.255 [−13.032; 17.742]	0.403	−0.305 [−6.217; 1.668]	0.108		
FU2	–	FU3	0.184 [−0.009; 0.715]	**0.014**	−0.236 [−5.156; 6.138]	0.614	−0.036 [−9.492; 3.074]	0.405		
FU2	–	FU4	0.220 [−0.150; 0.737]	**0.024**	−0.614 [−9.117; 14.376]	0.403	−0.127 [−9.476; 3.227]	0.204		
FU3	–	FU4	−0.020 [−0.328; 0.213]	0.838	−0.099 [−3.000; 7.114]	0.736	−0.146 [−1.373; 1.541]	0.659		

*p*-value is in bold if it reached statistical significance.

Decreasing follow-up totals correspond to patient termination of follow-ups before the end of the study.

**Figure 2:**
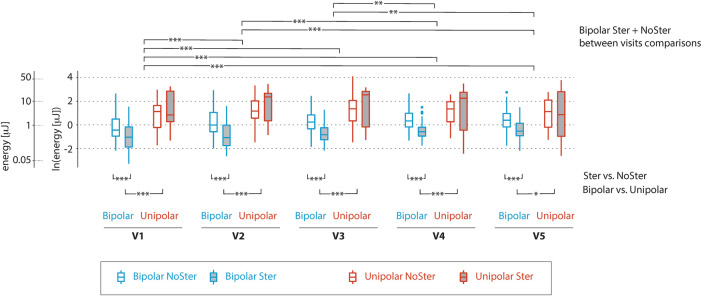
Graphical comparison of capture threshold pacing energies as they changed during individual visits. The patient group is divided into four boxplots for each visit (Bipolar-NSE blue without fill, Bipolar-SE blue with grey full, Unipolar-NSE red without fill and Unipolar-NSE red with grey fill). Each boxplot shows median and upper and lower quartiles, line extension (whiskers) reach the maximum value, at maximum 1.5 times the interquartile range, outliers are represented by dots. The capture threshold energy is shown on logarithmic scale. Statistically significant differences between groups differing in types of vectors and steroid eluting are shown below the boxplots, differences between visits for the group with the bipolar vectors without steroid eluting are at the top of the figure. In particular, the figure illustrates the differences in energies for bipolar and pseudo-unipolar vectors as well as the effect of steroid eluting in bipolar. The gradual dynamics of the increase in the pacing threshold for the bipolar vectors is particularly noticeable for the first three visits.

For better clarity of the development, the data was also processed in a graphic representation in graph—Figure 2.

Upon closer data processing, large differences in threshold energies were found between bipolar and pseudo-unipolar vectors. Therefore, the file was further divided according to these two modalities (Table 2).

### Bipolar vectors

As with the entire group, the statistical significance of changes in the pacing energies of bipolar (BIP) vectors between individual visits was also tested. They were calculated to be statistically significantly different using Friedman test (*p* < 0.001). Values after implantation (IMPL) increased by approximately 30% (*p* < 0.001) until discharge (FU1). By the first visit after stabilization of parameters (FU2), there was an insignificant increase of 13% (*p* = 0.219). The increase in threshold energy from implantation values (IMPL) to steady-state values (FU2) was a total of 48% (*p* < 0.001). In the next follow-up, there was a gradual statistically significant increase in threshold energies by 27% from FU2 (*p* = 0.004). The overall increase in the capture threshold energy from implantation to the fifth final control (FU4) was a statistically significant 88% (*p* < 0.001) (Tables 2).

### Pseudo-unipolar vectors

Using the Friedman test, it was calculated that there are no statistically significant differences in the value of the pseudo-unipolar vectors (*p* = 0.064). It was calculated that the values after measurement at implantation (IMPL) increased by approximately 9% (*p* = 0.011) to discharge (FU1). By the first visit after stabilization of the parameters (FU2) there was an insignificant increase of 23% (*p* = 0.762). The increase in the threshold energy from implantation values (IMPL) to stable values (FU2) was a total of 34% (*p* = 0.024). In the next follow-up, there was a gradual reduction of threshold energies by 24% from FU2 to FU4 (*p* = 0.122). Overall, the values from the measured implantation (IMPL) to the fifth final control (FU4) hardly changed at 3% (*p* = 0.463) (Tables 2, 3).

### Bipolar vs. pseudo-unipolar vectors

A statistically significant difference in thresholds for bipolar and pseudo-unipolar vectors was confirmed for all controls (Table 3). Mean values of the capture threshold energy were several times higher for pseudo-unipolar vectors (UNI) than bipolar ones (BI) when measured during all visits (Table 2).

### Bipolar vectors with steroid elution

The distribution according to the equipment of the pacing pole with a steroid is shown in Table 3. Bipolar (BI) and pseudo-unipolar vectors (UNI) were evaluated separately, because these two categories had large differences in the absolute values of the pacing energies.

Energies of bipolar steroid-eluting D1-X vectors (SE) were evaluated. Statistical significances of changes in pacing energies between individual visits were calculated. Using Friedman coefficient, the statistical significance of the measurement differences was verified (*p* = 0.006). Values after measurement at implantation (IMPL) decreased by approximately 3% (*p* = 0.748) until discharge (FU1). By the first visit after the stabilization of parameters (FU2), there was an insignificant increase of 27% (*p* = 0.226). The increase in the threshold energy from implantation values (IMPL) to stable values (FU2) was a total of 23% (*p* = 0.372). In the next follow-up, there was a gradual increase in the threshold energies by 36% from FU2 (*p* = 0.065). The overall increase in the capture threshold energy from implantation to the fifth final visit (FU4) was 68% (*p* = 0.007) (Tables 2, 3).

### Bipolar vectors without steroid elution

For bipolar vectors (BI) without steroid elution (NSE), there are significant differences between visits (Friedman, *p* < 0.001). After implantation (IMPL), there was a large increase of approximately 53% (*p* < 0.001) until discharge (FU1). By the first visit after the stabilization of parameters (FU2), there was a significant increase of 29% (*p* = 0.001). The increase in the threshold energy from implantation values (IMPL) to steady-state values (FU2) was almost double 98% (*p* < 0.001). In the next follow-up, there was a gradual increase in threshold energies by 17% from FU2 (*p* = 0.024). The overall increase in the capture threshold energy from implantation (IMPL) to the fifth final control (FU4) was 131% (*p* < 0.001). (Tables 2, 3).

### Bipolar vectors, steroid-eluted vs. steroid-free

When comparing the values of bipolar vectors with steroids (SE) and without steroids (NSE), there was always a statistically significant difference (Table 4, *p* < 0.001). Pacing pulse energy values of Steroid vectors (SE) were approximately 2-fold lower at implantation and this difference increased to approximately 2.5-fold over time compared to the vectors without steroids (NSE) (Table 4).

**Table 4:** Comparison of pacing energies for different modalities.

Independent samples *t*-test (Mann–Whitney *U*)						
	Part A		Part B		Part C	
	BI vs. UNI		BI. Ster vs. NoSter		UNI. Ster vs. NoSter	
	Difference Mean [95% CI]	*p*	Difference Mean [95% CI]	*p*	Difference Mean [95% CI]	*p*
IMPLANT	2.024 [1.206; 3.326]	**<.001**	0.291 [0.131; 0.485]	**<.001**	−1.182 [−15.27; 2.190]	0.502
Follow-up1	2.649 [1.598; 5.603]	**<.001**	0.629 [0.390; 0.975]	**<.001**	−2.630 [−12.116; 2.754]	0.502
Follow-up2	2.847 [1.096; 5.443]	**<.001**	0.682 [0.432; 1.089]	**<.001**	−4.235 [−13.971; 3.149]	0.564
Follow-up3	2.199 [0.707; 4.931]	**<.001**	0.798 [0.515; 1.161]	**<.001**	−3.691 [−13.288; 2.330]	0.533
Follow-up4	3.502 [0.194; 3.502]	**0.012**	0.692 [0.373; 1.072]	**<.001**	−0.226 [−17.480; 3.424]	0.845

*p*-value is in bold if it reached statistical significance.

### Pseudo-unipolar vectors with steroid elution

In the next step, the energies of pseudo-unipolar steroid-eluted vectors D1-X (UNI-SE) were evaluated. Using Friedman coefficient, the statistical significance of the measurement differences was verified (*p* = 0.878). Values after measurement at implantation (IMPL) increased by approximately 460% (*p* = 0.736) to discharge (FU1). By the first visit after the stabilization of parameters (FU2) there was a 19% increase (*p* = 1). The increase in the threshold energy from implantation values (IMPL) to stable values (FU2) was a total of 545% (*p* = 0.736). In the next follow-up, there was a gradual reduction of threshold energies by 53% from FU2 (*p* = 0.403). Overall, the capture threshold energy from implantation to the fifth final visit (FU4) in this category increased 2.5 times (256%, *p* = 0.614). No change showed statistical significance (Tables 2, 3).

### Pseudo-unipolar vectors without steroid elution

Furthermore, the energies of pseudo-unipolar vectors without Steroid elution (UNI-NSE) were evaluated. The statistical significance of changes in pacing energies by individual visits was calculated (Friedman, *p* = 0.061). The values after measurement at implantation (IMPL) increased by approximately 4% (*p* = 0.007) until discharge (FU1). By the first visit after stabilization of the parameters (FU2) there was a 22% increase (*p* = 0.731). The increase in the threshold energy from implantation values (IMPL) to steady-state values (FU2) was a total of 27% (*p* = 0.018). During subsequent follow-ups, there was a gradual decrease in threshold energies by 22% from FU2 (*p* = 0.204). Overall, the capture threshold energy from implantation to the fifth final control (FU4) practically did not change in this category; there was a decrease of 0.3% (*p* = 0.659). Only the acute change after implantation showed statistical significance (Tables 2, 3).

### Pseudo-unipolar vectors, steroid-eluted vs. steroid-free

When comparing the values of pseudo-unipolar vectors (UNI) with steroids (SE) and without them (NSE), there is no statistically significant difference (Table 4). Pacing pulse energy values of steroid vectors (SE) were about 2.5 times higher compared to vectors without steroids (NSE) during the entire observation, and this difference basically did not change during the further course—Table 4.

The graph above shows pairwise comparisons and average value of differences in the threshold pacing energy between individual visits (IMPL to FU4) for individual patient groups. The patient groups are listed in the header. If the Friedman test reached significance, the mean of individual differences with a 95% confidence interval is accompanied by a *p* value. If they reached significance in pairwise comparisons, individual *p* values of pairwise comparisons are in bold. The values show an increase in the capture threshold over time, especially in the first days and months after implantation (IMPL, FU1 and FU2).

The graph above shows a comparison of the threshold pacing energy for individual measurements (IMPL to FU4) between patients with pseudo-unipolar and bipolar vectors (Part A), Vectors with and without Steroid elution in bipolar (Part B) and pseudo-unipolar vectors (Part C). The mean difference with a 95% confidence interval is accompanied by a *p*-value, which is in bold if it reached statistical significance. The values show that the bipolar connection and Steroid eluting of the electrodes significantly reduce the threshold energy.

## Discussion

In published literature pertaining to capture threshold, there are studies dealing with the use of bipolar or pseudo-unipolar vectors for resynchronization treatment (8–10). So far, only limited results have been published on the long-term stability of pacing parameters of left ventricular leads (4).

The pacing vector was selected in the studied group based on criteria such as anatomical position of the lead, electrical position of the electrode, and the presence of contraindications such as phrenic nerve stimulation and an acceptable capture threshold. Furthermore, the acute effect of resynchronization therapy assessed by perioperative twelve-lead ECG was taken into account.

The measurements show that capture threshold energy in the whole group experienced a 43% increase on the first day after the lead placement (IMPL-FU1). Until the third measurement after 3 months, the value stabilized at 50% increase from the values during implantation (IMPL-FU2). The next measurement showed only a 19% increase to the total value, which was 80% higher than the implantation value (IMPL-FU4). Similar results are reported in the literature only for a smaller set of patients (4) and for right ventricular leads, where there are studies based on large sets and long-term follow-ups. Here, the value of the capture threshold energy is usually at least double compared to the implantation ones and increases slightly over time (2).

The results of this observational study further show that the use of bipolar vectors resulted in a significantly lower energy requirement compared to the use of pseudo-unipolar vectors, while maintaining the effect of biventricular pacing. Bipolar vectors were shown to have a significantly higher pacing impedance (*p* < 0.005) and a lower capture threshold (*p* < 0.005). The resulting energy consumed per pulse was thus 4–5 times higher for pseudo-unipolar vectors, which can subsequently lead to a faster depletion of the device battery. The above results suggest a preference for the bipolar vectors. This outcome can be also supported by the existing research comparing the effects of resynchronization treatment using bipolar vectors and pseudo-unipolar vectors. Based on published data, the QRS complex is significantly narrower when using the bipolar vector, given the cathode location (e.g., 135.1 ± 17.8 ms vs. 119.3 ± 14.5 ms—*p* < 0.01) (10, 11).

It needs to be reiterated that the presented study is observational. For this reason, it was not possible to measure every possible pacing vector during patients' visits and the reported results are limited to the used vectors. At the same time, there are no systemic biases involved in the sample selection process and, thus, the impact of potential confounders on the observed effect is minimized. Additionally, if for a given patient there were multiple pacing vectors with comparable resynchronization effects available, the one consuming the least amount of energy was selected. This selection process was a part of the study design, as one of the aims was to study the impact of pacing vectors on device longevity.

Finally, the data showed that Steroid eluting has an effect on the acute increase in the capture threshold after placing the lead endovascularly. For the Steroid-free electrodes in the case of bipolar pacing, there is an increase in pacing energies by 98% (IMPL-FU2). In case Steroid elution is used, the increase was only by 27% (IMPL-FU2). Further measurements showed that there was an increase in minimum pacing energies for Steroid-free vectors by 131% (IMPL-FU4) and a moderate increase for Steroid eluted vectors—by 68% (IMPL-FU4). The mentioned results correspond to the measurements with endocardially placed leads (2, 12). The potential influence of Steroids in the case of left ventricular leads was experimentally confirmed in an animal model as well (13).

That being said, it needs to be stated that the existing evidence points in the direction of a lesser observed steroid effect. Our study shows that in certation situations the effect can be more pronounced and delimits the conditions for making such an effect measurable.

### Limitations

When comparing the effect of Steroid eluting electrodes with pseudo-unipolar vector, the results did not reach significance. In the group of Steroid coated vectors (7 observations), the absence may be due to the low number of observations.

It is not possible to create a continuous time series from the measured data, including an acute increase in the capture threshold, because we used clinically defined measurement intervals. In the period of the acute threshold increase, we had only one value (FU1) (12–14). In the literature, it is stated that Steroid elution causes the differences in the development of the threshold energy only after a few days (12). In our case, the perioperative values and the same FU1 values one day after the implantation differ. The difference could be explained by a modern design of the electrode or a different type of connective tissue (venous endothelium vs. endocardium).

A single left ventricular lead model with three shapes from one manufacturer was used during the study.

## Conclusion

After the initial increase, leads for endovascular pacing of the left heart ventricle showed stable values of threshold pacing energies. The long-term effect and stability of these values did not differ substantially even when divided into pseudo-unipolar and bipolar configurations. The pseudo-unipolar vectors had a significantly higher threshold pacing energy, which may have an adverse effect on the battery life of the implanted device. In practice, bipolar vectors are preferred by default. The results of this study support this practice based on measurements from a relatively large patient sample. Furthermore, the study verified that the use of Steroid eluting has a significant positive effect even in endovascular pacing, not only in the acute phase after the placement of the left ventricular lead, but also in the long-term follow-ups.

## Data Availability

The raw data supporting the conclusions of this article will be made available by the authors, without undue reservation.
